# How Neuroscience Relates to Hearing Aid Amplification

**DOI:** 10.1155/2014/641652

**Published:** 2014-06-18

**Authors:** K. L. Tremblay, C. W. Miller

**Affiliations:** Department of Speech and Hearing Sciences, University of Washington, Seattle, WA 98105, USA

## Abstract

Hearing aids are used to improve sound audibility for people with hearing loss, but the ability to make use of the amplified signal, especially in the presence of competing noise, can vary across people. Here we review how neuroscientists, clinicians, and engineers are using various types of physiological information to improve the design and use of hearing aids.

## 1. Introduction

Despite advances in hearing aid signal processing over the last few decades and careful verification using recommended clinical practices, successful use of amplification continues to vary widely. This is particularly true in background noise, where approximately 60% of hearing aid users are satisfied with their performance in noisy environments [1]. Dissatisfaction can lead to undesirable consequences, such as discontinued hearing aid use, cognitive decline, and poor quality of life [2, 3].

**Figure 1:**
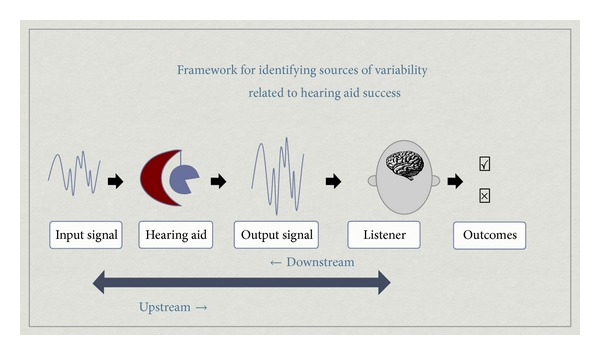
Framework for identifying sources of variability related to hearing aid success.

Many factors can contribute to aided speech understanding in noisy environments, including device centered (e.g., directional microphones, signal processing, and gain settings) and patient centered variables (e.g., age, attention, motivation, and biology). Although many contributors to hearing aid outcomes are known (e.g., audibility, age, duration of hearing loss, etc.), a large portion of the variance in outcomes remains unexplained. Even less is known about the influence interacting variables can have on performance. To help advance the field and spawn new scientific perspectives, Souza and Tremblay [4] put forth a simple framework for thinking about the possible sources in hearing aid performance variability. Their review included descriptions of emerging technology that could be used to quantify the acoustic content of the amplified signal and its relation to perception. For example, new technological advances (e.g., probe microphone recordings using real speech) were making it possible to explore the relationship between amplified speech signals, at the level of an individual's ear, and the perception of those same signals. Electrophysiological recordings of amplified signals were also being introduced as a potential tool for assessing the neural detection of amplified sound. The emphasis of the framework was on signal audibility and the ear-to-brain upstream processes associated with speech understanding. Since that time, many new directions of research have emerged, as has an appreciation of the cognitive resources involved when listening to amplified sounds. We therefore revisit this framework when highlighting some of the advances that have taken place since the original Souza and Tremblay [4] article (e.g., SNR, listening effort, and the importance of outcome measures) and emphasize the growing contribution of neuroscience (Figure 1).

## 2. Upstream, Downstream, and Integrated Stages

A typical example highlighting the interaction between upstream and downstream contributions to performance outcomes is that involving the cocktail party. The cocktail party effect is the phenomenon of a listener being able to attend to a particular stimulus while filtering out a variety of competing stimuli, similar to partygoer focusing on a single conversation in a noisy room [5, 6]. The ability of a particular individual to “tune into” a single voice and “tune out” all that is coming out of their hearing aid is also an example of how variables specific to the individual can also contribute to performance outcomes.

When described as a series of upstream events that could take place in someone's everyday life, the* input signal* refers to the acoustic properties of the incoming signal and/or the context in which the signal is presented. It could consist of a single or multiple talkers; it could be an auditory announcement projected overhead from a loudspeaker at the airport, or it could be a teacher giving homework instructions to children in a classroom. It has long been known that the ability to understand speech can vary in different types of listening environments because the signal-to-noise ratio (SNR) can vary from −2 dB, when in the presence of background noise outside the home, to +9 dB SNR, a level found inside urban homes [7]. Support for the idea that environmental SNR may influence a person's ability to make good use of their hearing aids comes from research showing that listeners are more dissatisfied and receive less benefit with their aids in noise than in quiet environments (e.g., [1, 8, 9]). From of a large-scale survey, two of the top three reasons for nonadoption of aids were that aids did not perform well in noise (48%) and/or that they picked up background sounds (45%; [10]). And of the people who did try aids, nearly half of them returned their aids due to lack of perceived benefit in noise or amplification of background noise. It is therefore not surprising that traditional hearing aid research has focused on hearing aid engineering in attempt to improve signal processing in challenging listening situations, so that optimal and audible signals can promote effective real-world hearing.

The next stage emphasizes the contribution ofthe* hearing aid* and how it modifies the acoustic signal (e.g., compression, gain and advanced signal processing algorithms). Examples include the study of real-world effectiveness of directional microphone and digital noise reduction features in hearing aids (e.g., [11, 12]). Amplification of background noise is one of the most significant consumer-based complaints associated with hearing aids, and directional hearing aids can improve the SNR of speech occurring in a noisy background (e.g., [13, 14]). However, these findings in the laboratory may not translate to perceived benefit in the real world. When participants were given a four-week take-home trial, omnidirectional microphones were preferred over directional microphones [15]. Over the past several decades, few advances in hearing aid technology have been shown to result in improved outcomes (e.g., [9, 16]). Thus, attempts at enhancing the quality of the signal do not guarantee improved perception. It suggests that something, in addition to signal audibility and clarity, contributes to performance variability.

What is received by the individual's auditory system is not the signal entering the hearing aid but rather a modified signal leaving the hearing aid and entering the ear canal. Therefore, quantification of the signal at the* output of the hearing aid* is an important and necessary step to understanding the biological processing of amplified sound. Although simple measures of the hearing aid output (e.g., gain for a given input level) in a coupler (i.e., simulated ear canal) have been captured for decades, current best practice guidelines highlight the importance of measuring hearing aid function in the listener's own ear canal. Individual differences in ear canal volume and resonance and how the hearing aid is coupled to an individual's ear can lead to significant differences in ear canal output levels [17]. Furthermore, as hearing aid analysis systems become more sophisticated, we are able to document the hearing aid response to more complex input signals such as speech or even speech and noise [18], which provides greater ecological validity than simple pure tone sweeps. In addition, hearing aid features can alter other acoustic properties of a speech signal. For example, several researchers have evaluated the effects of compression parameters on temporal envelope or the slow fluctuations in a speech signal [19–23], spectral contrast or consonant vowel ratio [20, 24–26], bandwidth [24], effective compression ratio [23, 24, 27], dynamic range [27], and audibility [24, 27, 28]. For example, as the number of compression channels increases, spectral differences between vowel formants decrease [26], the level of consonants compared to the level of vowels increases [29], and dynamic range decreases [27]. Similarly, as compression time constants get shorter, the temporal envelope will reduce/smear [20, 21, 23] and the effective compression ratio will increase [27]. A stronger compression ratio has been linked to greater temporal envelope changes [21, 23]. Linear amplification may also create acoustic changes, such as changes in spectral contrast if the high frequencies have much more gain than the low frequencies (e.g., [24]). The acoustic changes caused by compression processing have been linked to perceptual changes in many cases [19–22, 24, 26, 30]. In general, altering compression settings (e.g., time constants or compression ratio) modifies the acoustics of the signal and the perceptual effects can be detrimental. For this reason, an emerging area of interest is to examine how frequency compression hearing aid technology affects the neural representation and perception of sound [31].

**Figure 2:**
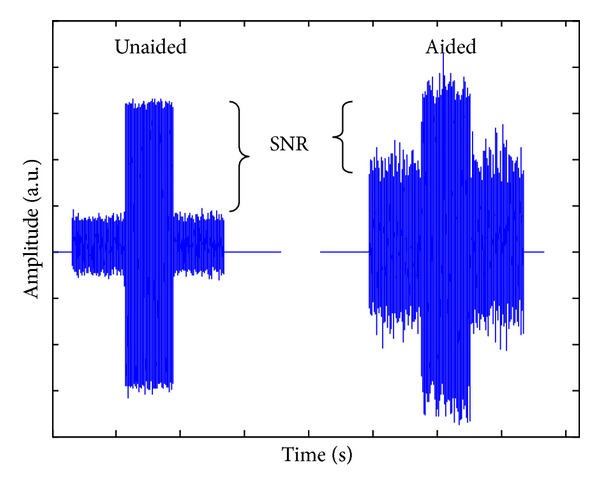
Time waveforms of in-the-canal acoustic recordings for one individual. The unaided (left) and aided (right) conditions are shown together. Signal output as measured at the 1000 Hz centered 1/3 octave band was approximately equivalent at 73 and 74 dB SPL for the unaided and aided conditions. However, noise levels in the same 1/3 octave band were approximately 26 and 54 dB SPL, demonstrating the significant change in SNR.

**Figure 3:**
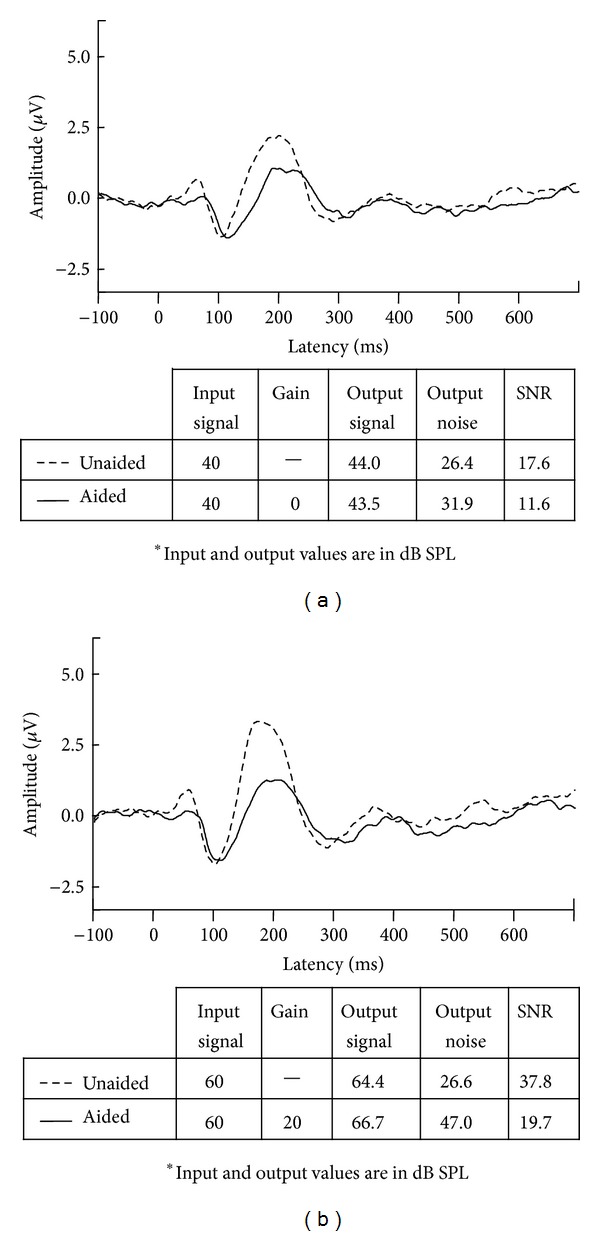
Two examples showing grand mean CAEPs recorded with similar mean output signal levels. Panels: (a) 40 dB input signals and (b) 60 dB input signals show unaided and aided grand mean waveforms evoked with corresponding in-the-canal acoustic measures. Despite similar input and output signal levels, unaided and aided brain responses are quite different. Aided responses are smaller than unaided responses, perhaps because the SNRs are poorer in the aided condition.

Characteristics of the* listener* (e.g., biology) can also contribute to a person's listening experience. Starting with bottom-up processing, one approach in neuroscience has been to model the auditory-nerve discharge patterns in normal and damaged ears in response to speech sounds so that this information can be translated into new hearing aid signal processing [32, 33]. The impact of cochlear dead regions on the fitting of hearing aids is another example of how biological information can influence hearing aid fitting [34]. Further upstream, Willott [49] established how aging and peripheral hearing loss affects sound transmission, including temporal processing, at higher levels in the brain. For this reason, brainstem and cortical evoked potentials are currently being used to quantify the neural representation of sound onset, offset and even speech envelope, in children and adults wearing hearing aids, to assist clinicians with hearing aid fitting [36–39]. When evoked by different speech sounds at suprathreshold levels, patterns of cortical activity (e.g., P1-N1-P2—also called acoustic change responses (ACC)) are highly repeatable in individuals and can be used to distinguish some sounds that are different from one another [4, 37]. Despite this ability, we and others have since shown that P1-N1-P2 evoked responses do not reliably reflect hearing aid gain, even when different types of hearing aids (analog and digital) and their parameters (e.g., gain and frequency response) are manipulated [40–45]. What is more, the signal levels of phones when repeatedly presented in isolation to evoke cortical evoked potentials are not the same as hearing aid output levels when phonemes are presented in running speech context [46]. These examples are provided because they reinforce the importance of examining the output of the hearing aid. Neural activity is modulated by both endogenous and exogenous factors and, in this example, the P1-N1-P2 complex was driven by the signal-to-noise ratio (SNR) of the amplified signal.  Figure 2 shows the significant effect hearing aid amplification had on SNR when Billings et al. [43] presented a 1000 Hz tone through a hearing aid. Hearing aids are not designed to process steady-state tones, but results are similar even when naturally produced speech syllables were used [37]. Acoustic waveforms, recorded in-the-canal, are shown (unaided = left; aided=right). The output of the hearing aid, as measured at the 1000 Hz centered 1/3 octave band, was approximately equivalent at 73 and 74 dB SPL for unaided and aided conditions. Noise levels in that same 1/3 octave band, however, approximated 26 dB in the unaided condition and 54 dB SPL in the aided condition. Thus SNRs in the unaided and aided conditions, measured at the output of the hearing aid, were very different, and time-locked evoked brain activity shown in Figure 3 was influenced more by SNR than absolute signal level. Most of these SNR studies have been conducted in normal hearing listeners and thus the noise was audible, something unlikely to occur at some frequencies if a person has a hearing loss. Nevertheless, noise is always present in an amplified signal and contributors may range from amplified ambient noise to circuit noise generated by the hearing aid. It is therefore important to consider the effects of noise, among the many other modifications introduced by hearing aid processing (e.g., compression) on evoked brain activity. This is especially important because commercially available evoked potential systems are being used to estimate aided hearing sensitivity in young children [47].

What remains unclear is how neural networks process different SNRs, facilitate the suppression of unwanted competing signals (e.g., noise), and process simultaneous streams of information when people with hearing loss wear hearing aids. Individual listening abilities have been attributed to variability involving motivation, selective attention, stream segregation, and multimodal interactions, as well as many other cognitive contributions [48]. It can be mediated by the biological consequences of aging and duration of hearing loss, as well as the peripheral and central effects of peripheral pathology (for reviews see [44, 49]). Despite the obvious importance of this stage and the plethora of papers published each year on the topics of selective attention, auditory streaming, object formation, and spatial hearing, the inclusion of people with hearing loss and who wear hearing aids remains relatively slim.

Over a decade ago, a working group that included scientists from academia and industry gathered and discussed the need to include central factors when considering hearing aid use [50] and since then there has been increased awareness about including measures of cognition, listening effort, and other top-down functions when discussing rehabilitation involving hearing aid fitting [51]. However, finding universally agreed upon definitions and methods to quantify cognitive function remains a challenge. Several self-report questionnaires and other subjective measures have evolved to measure listening effort, for example, but there are also concerns that self-report measures do not always correlate with objective measures [52, 53]. For this reason, new explorations involving objective measures are underway.

There have been tremendous advances in technology that permit noninvasive objective assessments of sensory and cognitive function. With this information it might become possible to harness cognitive resources in ways that have been previously unexplored. For example, it might become possible to use brain measures to guide manufacturer designs. Knowing how the auditory system responds to gain, noise reduction, and/or compression circuitry could influence future generations of biologically motivated changes in hearing aid design. The influence of brain responses is especially important with current advances in hearing aid design featuring binaural processing, which involve algorithms making decisions based on cues received from both hearing aids. Returning to the example of listening effort, pupillometry [54], an objective measure of pupil dilation, and even skin conductance (EMG activity; [55]) are being explored as an objective method for quantifying listening effort and cognitive load. Other approaches include the use of EEG and other neuropsychological correlates of auditive processing for the purpose of setting a hearing device by detecting listening effort [56]. In fact, there already exist a number of existing patents for this purpose by hearing aid manufacturers such as Siemens, Widex, and Oticon, to name a few. These new advances in neuroscience make it clear that multidisciplinary efforts that combine neuroscience and engineering and are verified using clinical trials are innovative directions in hearing aid science. Taking this point one step further, biological codes have been used to innervate motion of artificial limbs/prostheses, and it might someday be possible to design a hearing prosthesis that includes neuromachine interface systems driven by a person's listening effort or attention [57–59]. Over the last decade, engineers and neuroscientists have worked together to translate brain-computer-interface systems from the laboratory for widespread clinical use, including hearing loss [60]. Most recently, eye gaze is being used as a means of steering directional amplification. The visually guided hearing aid (VGHA) combines an eye tracker and an acoustic beam-forming microphone array that work together to tune in the sounds your eyes are directed to while minimizing others [61]. The VGHA is a lab-based prototype whose components connect via computers and other equipment, but a goal is to turn it into a wearable device. But, once again, the successful application of future BCI/VGHA devices will likely require interdisciplinary efforts, described within our framework, given that successful use of amplification involves more than signal processing and engineering.

If a goal of hearing aid research is to enhance and empower a person's listening experience while using hearing aids, then a critical metric within this framework is the outcome measure. Quantifying a person's listening experience using a hearing aid as being positive [*✓*] or negative [*✗*] might seem straight forward, but decades of research on the topic of outcome measures show this is not the case. Research aimed at modeling and predicting hearing aid outcome [9, 62] shows that there are multiple variables that influence various hearing aid outcomes. A person's age, their expectations, and the point in time in which they are queried can all influence the outcome measure. The type of outcome measure, self-report or otherwise, can also affect results. It is for this reason that a combination of measures (e.g., objective measures of speech-understanding performance; self-report measures of hearing aid usage; and self-report measures of hearing aid benefit and satisfaction) is used to characterize communication-related hearing aid outcome. Expanding our knowledge about the biological influences on speech understanding in noise can inspire the development of new outcome measures that are more sensitive to a listener's perception and to clinical interventions. For example, measuring participation in communication may assess a listener's use of their auditory reception on a deeper level than current outcomes asking how well speech is understood in various environments [63], which could be a promising new development in aided self-report outcomes.

## 3. Putting It All Together

Many factors can contribute to aided speech understanding in noisy environments, including device centered (e.g., directional microphones, signal processing, and gain settings) and patient centered variables (e.g., age, attention, motivation, and biology). The framework (Figure 1) proposed by Souza and Tremblay [4] provides a context for discussing the multiple stages involved in the perception of amplified sounds. What is more, it illustrates how research aimed at exploring one variable in isolation (e.g., neural mechanisms underlying auditory streaming) falls short of understanding the many interactive stages that are involved in auditory streaming in a person who wears a hearing aid. It can be argued that it is necessary to first understand how normal hearing ear-brain systems stream, but it can also be argued that interventions based on normal hearing studies are limited in their generalizability to hearing aid users.

A person's self-report or aided performance on an outcome measure can be attributed to many different variables illustrated in Figure 1. Each variable (e.g., input signal) could vary in different ways. One listener might describe themselves as performing well [*✓*] when the input* signal* is a single speaker in moderate noise conditions, provided they are paying* attention* to the speaker while using a hearing aid that makes use of a* directional microphone*. This same listener might struggle [*✗*] if this single speaker is a lecturer in the front of a large classroom who paces back and forth across the stage and intermittently speaks into a microphone. In this example, changes in the quality and direction of a single source of input may be enough to negatively affect a person's use of sound upstream because of a reduced neural capacity to follow sounds when they change in location and in space. This framework and these examples are overly simplistic, but they are used to emphasize the complexity and multiple interactions that contribute to overall performance variability. We also argue that it is overly simplistic for clinicians and scientists to assume that explanations of performance variability rest solely one stage/variable. For this reason, interdisciplinary research that considers the contribution of neuroscience as an important stage along the continuum is encouraged.

The experiments highlighted here serve as examples to show how far, and multidisciplinary, hearing aid research has come. Since the original publication of Souza and Tremblay [4], advances have been made on the clinical front as shown through the many studies aimed at using neural detection measures to assist with hearing aid fitting. And it is through neuroengineering that that next generation of hearing prostheses will likely come.
